# Material Extrusion Filament Width and Height Prediction via Design of Experiment and Machine Learning

**DOI:** 10.3390/mi14112091

**Published:** 2023-11-12

**Authors:** Xiaoquan Shi, Yazhou Sun, Haiying Tian, Puthanveettil Madathil Abhilash, Xichun Luo, Haitao Liu

**Affiliations:** 1 Department of Mechanical Engineering and Automation, Harbin Institute of Technology, Harbin 150001, China; xiaoquanhit@163.com (X.S.); thy0567@163.com (H.T.); 2 Centre for Precision Manufacturing, Department of Design, Manufacturing & Engineering Management, University of Strathclyde, Glasgow G1 1XJ, UK; abhilash.p-m@strath.ac.uk (P.M.A.); xichun.luo@strath.ac.uk (X.L.)

**Keywords:** material extrusion 3D printing, processing parameters, filament dimension, design of experiment, machine learning

## Abstract

The dimensions of material extrusion 3D printing filaments play a pivotal role in determining processing resolution and efficiency and are influenced by processing parameters. This study focuses on four key process parameters, namely, nozzle diameter, nondimensional nozzle height, extrusion pressure, and printing speed. The design of experiment was carried out to determine the impact of various factors and interaction effects on filament width and height through variance analysis. Five machine learning models (support vector regression, backpropagation neural network, decision tree, random forest, and K-nearest neighbor) were built to predict the geometric dimension of filaments. The models exhibited good predictive performance. The coefficients of determination of the backpropagation neural network model for predicting line width and line height were 0.9025 and 0.9604, respectively. The effect of various process parameters on the geometric morphology based on the established prediction model was also studied. The order of influence on line width and height, ranked from highest to lowest, was as follows: nozzle diameter, printing speed, extrusion pressure, and nondimensional nozzle height. Different nondimensional nozzle height settings may cause the extruded material to be stretched or squeezed. The material being in a stretched state leads to a thin filament, and the regularity of processing parameters on the geometric size is not strong. Meanwhile, the nozzle diameter exhibits a significant impact on dimensions when the material is in a squeezing state. Thus, this study can be used to predict the size of printing filament structures, guide the selection of printing parameters, and determine the size of 3D printing layers.

## 1. Introduction

Additive manufacturing (AM), also known as rapid prototyping, 3D printing, and freeform fabrication, is an advanced manufacturing method founded upon 3D model data. This method constructs intricate structures or parts by depositing materials layer-by-layer [1,2]. In contrast to traditional manufacturing technologies, AM exhibits significant advantages, including its capacity to handle intricate structures, low waste generation, and heightened production efficiency [3]. Presently, massive methods for AM are prevalent, including vat photopolymerization [4], material inkjet [5], binder jetting [6], powder bed fusion [7], and direct energy deposition [8], among others. Material extrusion, also known as direct ink writing (DIW) or robot-casting, is a processing technology that uses pneumatic pressure or mechanical loads as driving forces to extrude a semi-solid material from a nozzle, then solidifies the extruded material and bonds it to the previously extruded material to form a solid structure [9,10]. The processed materials are referred to as ‘inks’, which typically manifest as viscoelastic non-Newtonian fluids characterized by shear thinning behavior [11,12]. The material viscosity decreases, causing it to flow out of the nozzle, when subjected to high shear strain rates. Subsequent to deposition, the viscosity recovers at a zero shear force and the material maintains its shape. This processing method shows good material compatibility, simple operation, and an economical equipment cost [13,14,15]. It has also been widely applied in the processing of flexible robots [16,17], wearable sensors [18,19], tissue engineering [20,21], electronic components [22,23], and food materials [24,25], exhibiting its huge application potential across diverse domains.

The success of material extrusion and the dimension of extruded filaments are subject to the influence of various process parameters, including extrusion pressure, printing speed, nozzle diameter, printing nozzle height, and printing temperature, among others. Materials with different viscosities exhibit their own ‘printability windows’ during printing. Only with reasonable printing parameters can the material achieve a uniform and stable extrusion. Additionally, the width and height of the printed filament under different process parameters are quite different. Effectively foreseeing the geometric dimensions of the printed line structure can be used to guide the slicing of 3D models and improve the manufacturing performance of 3D structures [25]. The determination of extrusion parameters tends to rely on experiential approaches or extensive trial and error tests, often entailing a significant time and labor investment [26].

Establishing mathematical models and theoretical calculations is a method for determining process parameters. Udofia and Zhou [27] summarized numerous parameters influencing filament width and height, encompassing extrusion pressure, printing speed, nozzle diameter, nozzle height, nozzle length, ink viscosity, surface tension surface property (e.g., contact angle, θ), etc. However, the interplay of these factors is intricate, and an integral functional equation describing the above variables has not yet been established. To streamline a model, a functional relationship linking the geometric dimensions of the printed line with the extrusion flow rate and printing speed should be devised, grounded in the extruded material’s adherence to the law of volume conservation [28,29]. However, this volume conservation model would exclusively pertain to filaments of a uniform and stable structure. The volumetric flow rate of printed ink emerges as a pivotal factor in the calculation process yet the control of the flow rate in pneumatic extrusion 3D printing poses challenges, given its intrinsic difficulty. Although plunger extrusion allows for flow rate adjustment, the viscoelastic nature of the ink, coupled with its compressibility, introduces inaccuracies in flow control [13,20].

Numerous parameters wield influence over the dimensional attributes of 3D printing structures, and their distribution spans a broad spectrum. The design of experiment (DoE) has become a powerful tool for studying the relationship between the printing structure size and various factors [30]. As a statistical analysis method, factorial design can reveal the degree of influence of printing parameters on response variables through statistical results [31]. Zhang et al. [22] investigated the effects of extrusion pressure, nozzle head height, and substrate moving speed on printed filament width by orthogonal experimental design. The results of the range analysis unveiled a descending hierarchy of effects for these three factors: printing speed, extrusion pressure, and nozzle height. Caputo et al. [31] performed an exploration of the surface roughness of fused filament fabrication (FFF) parts by the DoE approach; the analysis of variance and Pareto chart clearly showed that the nozzle temperature had a significant impact.

Machine learning constitutes yet another avenue for exploring the intricate nexus between the dimensions of 3D printing structures and their process parameters. It is a branch of artificial intelligence that focuses on developing statistical models and algorithms, enabling computers to learn adaptively from existing data and evolve without hard coding [32]. Machine learning has been successfully applied in various fields such as healthcare, energy, materials, and manufacturing [33,34,35,36]. Well-regarded machine learning methods include the artificial neural network (ANN), support vector machine (SVM), decision tree (DT), random forest (RF), K-nearest neighbors (KNN), and extreme gradient boost (XGBoost), among others. A comprehensive overview of machine learning’s applications in additive manufacturing, including predictions of mechanical properties, dimensional attributes, defect detection, and in situ monitoring, has been meticulously presented in Ref. [9]. Machine learning techniques have also been used to optimize the design parameters of 3D-printed composite layers [37,38,39]. Goh et al. [37] used neural networks and genetic algorithms to predict the shore hardness and compressive modulus, outperforming the surface response method by 3.5%. McGregor et al. [40] selected the SVM model to study the relationship between part geometry, quality, and different printing parameters. Subsequent to training, the model attained a root mean squared error (*RMSE*) of 53 µm, demonstrated a feature classification accuracy of 95%, and achieved a commendable part classification accuracy of 81%. Chen et al. [41] employed the ANN method to unravel the relationship between the printing pressure, nozzle height, and printing speed of SiC slurries and the printed filament width and height, yielding a predictive relative error of 0.045 for width and 0.100 for height. Ma et al. [42] chose an RF regression algorithm to predict the printing line width and height using a dataset of material viscosity and printing parameters, showcasing a predicted coefficient of determination (*R*^2^) ranging from 0.93 to 0.94. Machine learning is mainly aimed at data processing and analysis, is capable of discovering trends in large and nonlinear datasets, and reveals previously unknown or unclear relationships in high-dimensional data [40]. Given the divergence in model performance, scholars often undertake comparisons across various models while addressing the same problem and employing identical datasets. Ali et al. [32] selected Gaussian process regression models, DT regression models, SVM models, and XGBoost regression models to predict the tensile, compressive, and flexural strength of 3D-printed concrete materials, and their SVM model demonstrated the most favorable performance. Similarly, Wang et al. [30] studied the drug loading efficiency of 3D-printed drugs using five methods: DT, RF, KNN, XGBoost, and a light gradient boosting machine (LightGBM), with the DT model emerging as the top performer.

In this study, we present an investigation into the interplay between the geometric dimensions of pneumatic extrusion filament structures and printing parameters. Barium titanate/polydimethylsiloxane (BaTiO_3_/PDMS), which is a representative printing ink with shear thinning characteristics, was selected as the printing material. Due to its excellent dielectric properties, this material finds widespread utility in domains such as flexible sensors [43], energy storage materials [44], and terahertz technology [45]. DoE and machine learning methods were used to systematically evaluate the relationship between the printed filament dimension and main process parameters, including the extrusion pressure, nozzle diameter, printing speed, and nondimensional nozzle height. The significance of each factor was then studied through DoE statistical analysis. Five machine learning methods, including support vector regression (SVR), back-propagation neural network (BPNN), DT, RF, and KNN, were used to establish a model that illuminates the nexus between the geometric dimensions of printed filaments and printing parameters. Among these approaches, we identified the BPNN model as the most proficient performer, which we then leveraged to dissect the influence of varied process parameters on filament width and height. This analysis extended to deciphering the underlying patterns of influence, thereby offering guidance in the judicious selection of process parameters.

## 2. Materials and Methods

### 2.1. Three-Dimensional Printing and Dimension Measurement

BaTiO_3_ powder was purchased from Aladdin (Shanghai, China). PDMS Sylgard 184 and SE 1700 were obtained from Dow Corning. Before preparing composite materials, the base and curing agents of PDMS Sylgard184 and SE1700 were mixed in a weight ratio of 10:1. Subsequently, BaTiO_3_ powder, PDMS Sylgard184, and SE1700 were combined in a mass ratio of 3:4:8 for use as the printing material. To create the ink, the composite mixture was stirred at 2000 r/min within an ice–water bath for 15 min. This was followed by a 30-min vacuum treatment to eliminate any entrapped bubbles resulting from the stirring process. The filament structures were fabricated using an extrusion-based 3D printer. Different printing parameters were selected, which are described in the DoE study section and the machine learning section. After printing, the materials were cured in an oven at 80 °C for 6 h. The filament width and height were measured using a white light interferometer (Optical Surface Profile, Zygo, NewViewTM 8200 Series, Zygo Corporation, Middlefield, CT, USA).

### 2.2. DOE Study

Numerous factors intricately influence extrusion performance, with each parameter exhibiting a broad distribution range. We used the fractional factor design method in our experiment. This statistical analysis technique enables the systematic exploration of the impact of diverse process parameters on the dependent variable. In this study, we selected four process parameters: nozzle diameter, nondimensional nozzle height, extrusion pressure, and printing speed as factors for factorial design. Among them, the nondimensional nozzle height refers to the ratio of the nozzle height from the substrate to the nozzle diameter. We set three levels for each of these four factors and the DoE was carried out by Minitab 20 software, obeying the following two rules: the frequency of each factor at each level is the same and the frequency of any combination of two factors at any level is equal. The process parameters used in the experiment are shown in Table 1. After measuring the printed geometric dimensions of each parameter, we conducted an analysis of variance (ANOVA) to determine the magnitude of the interaction between the filament width and height and various factors.

We performed polynomial regression fitting to establish the relationship between the filament width and height, focusing specifically on parameters with a substantial impact. To increase the diversity of the data and enhance the precision of the fitting process and machine learning model, we conducted an additional set of 43 printing experiments. We kept three levels for the nozzle diameter and added another two levels for the other three factors. A comprehensive full-factor table was compiled, from which we randomly selected 43 parameters for the purpose of printing with the help of Minitab software. The detailed information on these selected parameters is presented in Table S1 (Supplementary Material). We used a total of 70 groups of parameters for regression and evaluated the fitting performance by calculating the coefficient of determination.

### 2.3. Machine Learning

**Figure 1:**
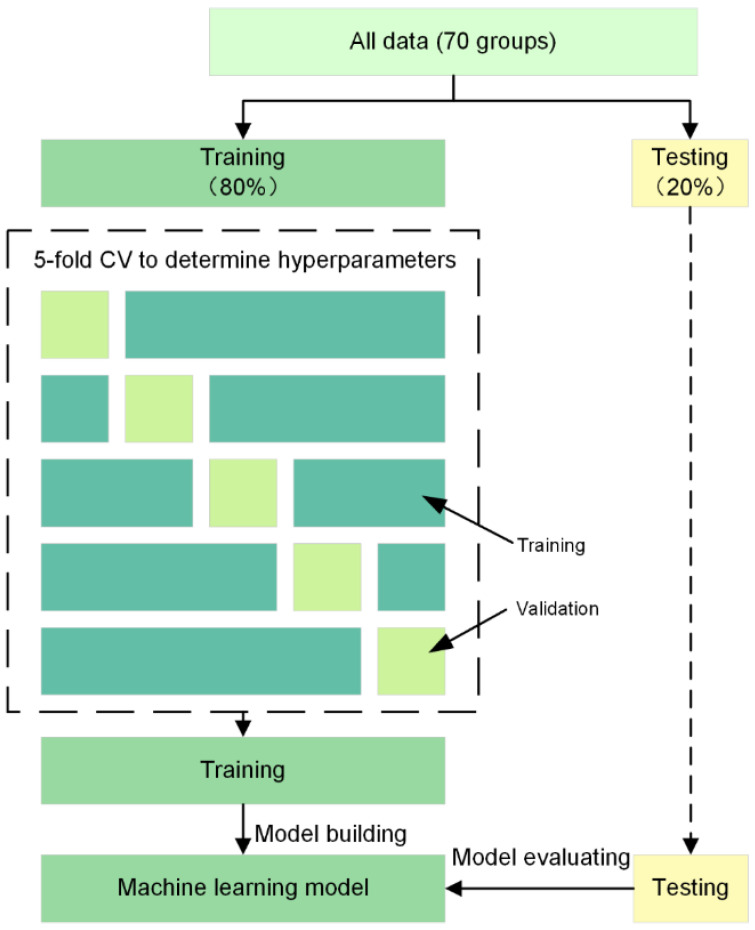
Method for training and testing the machine learning models with 5-fold cross-validation.

We employed five different machine learning methods, namely, SVR, BPNN, DT, RF, and KNN, for prediction. For training and testing, we used a dataset containing 70 groups of data, with 20% of the data used as the test set to evaluate the accuracy of the model’s prediction of unknown data. Given the limited size of the dataset, to obtain more reliable results, we adopted a 5-fold cross-validation strategy to ascertain the optimal hyperparameters for each model. During each calculation, four subsets were utilized for training the model, while the remaining subset was reserved for validation purposes. Hyperparameters with the smallest average root mean square error (*RMSE*) were chosen as the best ones and were selected for model training. This process is shown in Figure 1. All machine learning algorithms were implemented using Jupyter Notebook 6.5.4, Python version 3.8.12, and were constructed using the scikit-learning package. Given the disparate value ranges of the input variables, a standardization procedure was employed to mitigate the potential influence of these variations on the model outcomes. We standardized the four input parameters by adjusting the data to a distribution with a mean of 0 and a variance of 1. This approach was adopted to promote a balanced impact of distinct variables on the model. The calculation formula is provided below:

(1) $$x_{\mathrm{scale}}=\frac{x-\mu }{S}$$

where, *x* and *x*_scale_ are the data before and after standardization, and *μ* and *S* refer to the mean and variance of the data.

Three indicators were used for model performance evaluation, namely, root mean square error (*RMSE*), mean absolute error (*MAE*), and the coefficient of determination (*R*^2^). The formulae for calculating these evaluation metrics are provided below [46,47]:

(2) $$RMSE=\sqrt{\frac{1}{m}\sum_{i=1}^{m}{\left(f_{i}-y_{i}\right)}^{2}}$$

(3) $$MAE=\frac{1}{m}\sum_{i=1}^{m}\left|f_{i}-y_{i}\right|$$

(4) $$R^{2}=1-\frac{\sum_{i=1}^{m}{\left(f_{i}-y_{i}\right)}^{2}}{\sum_{i=1}^{m}{\left(y_{i}-\bar{y}\right)}^{2}}$$

where *f*_i_ and *y*_i_ refer to the prediction value and the true value, $\bar{y}$ is the mean of true value, and *m* is the total number of samples.

## 3. Results and Discussion

### 3.1. DOE and ANOVA Results

**Figure 2:**
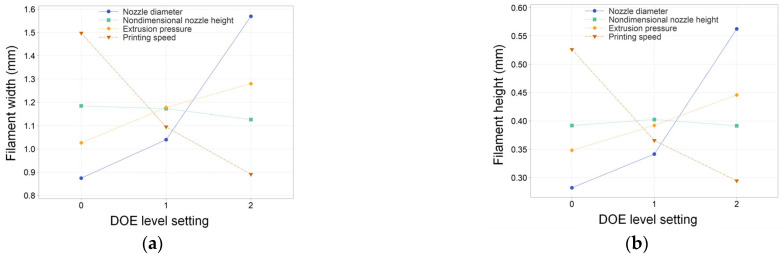
(**a**) The average filament width for each printing parameter at each level value and (**b**) the average filament height values for each printing parameter at each level value.

A filament can be fabricated smoothly and uniformly by using the given printing parameters, which proves that the selected parameters have good printing performance. The average values of the printed filament width and height under distinct parameters are presented in Table S2 (Supplementary Material). The span of the printed filament widths ranged from 0.3749 mm to 2.2892 mm, while the filament heights ranged from 0.1496 mm to 0.8541 mm. It can be seen that the printing parameters have a significant influence on the size of the filament. The interaction plots containing the average filament width and height values of the four process parameters at each of the three levels are shown in Figure 2. The figure reveals consistent rules in filament width and height variation as the four factors undergo changes, i.e., an increased nozzle diameter or pressure yields an augmented width and height, whereas an elevated printing speed corresponds to a diminished width and height. The impact of nozzle diameter and printing speed on the filament’s geometric dimensions surpasses that of printing pressure. When the nozzle diameter increased from 0.33 mm to 0.51 mm, the average width increased from 0.8745 mm to 1.5691 mm, and the average height increased from 0.2820 mm to 0.5624 mm. Similarly, an increase in printing speed from 2.5 mm/s to 7.5 mm/s elicits a reduction in the line width from 1.4973 mm to 0.8910 mm and line height from 0.5261 mm to 0.2944 mm. The effect of the nondimensional nozzle height on filament dimensions is slightly different: the line width decreased with the increase in the nondimensional nozzle height, while the line height experienced an initial increase followed by a subsequent decrease. Generally, the line height should also decrease with the increase in the nondimensional nozzle height. However, during printing, an excessively low nozzle height leads to material squeezing, causing it to flow in the normal direction along the printing direction [48,49], resulting in the actual filament height falling below the ideal state, as depicted by the green line in Figure 2b. Overall, the impact of the nondimensional nozzle height on geometric dimensions is notably smaller in comparison to the other three factors. Nondimensional nozzle height is used to describe the nozzle-to-substrate distance instead of the nozzle height by many scholars [50,51]. This research adopts the nondimensional nozzle height for two primary reasons: firstly, the variation range of nozzle diameter in the study is large, and fixed height values will lead to different printing states (material stretching or squeezing) for different nozzle diameters, which weakens the statistical regularity; secondly, the range of reasonable nozzle heights varies with different nozzle diameters, introducing complexity to the level setting.

**Figure 3:**
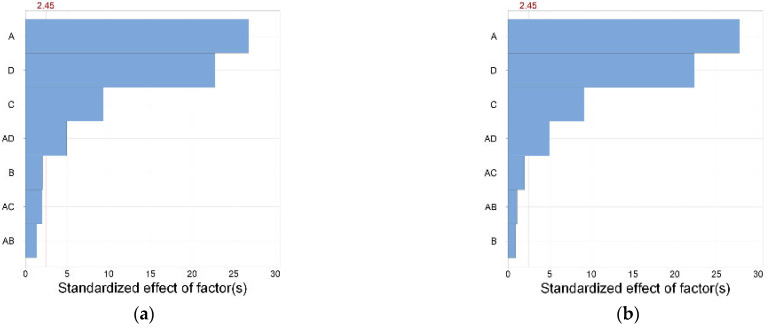
Pareto chart of the standardized effect of the (**a**) filament width and (**b**) filament height.

ANOVA serves as a significance testing method used to compare the mean differences across multiple samples. In this study, Minitab software was used to perform significance tests on the four response variables, with a significance level of 95% (α = 0.05). Table 2 and Table 3 show the results of the ANOVA for filament width and line height, and Figure 3a,b is the Pareto chart. The critical value of the standardization effect is 2.45, calculated by Minitab software. When the standardization effect is greater than this value, the *p*-value is less than 0.05, indicating this factor’s significant effect. Larger bars within the chart signify a greater statistical significance and correspondingly smaller *p*-values [30]. It can be observed from the Pareto chart that the impact of response variables on the line width and line height is very similar. The factors that have a significant impact ranked in order of their degree of influence are the nozzle diameter, printing speed, extrusion pressure, and nozzle diameter × printing speed; the former two factors wield a significantly greater influence on the response variables than the latter two. From the ANOVA results in Table 2 and Table 3, it can be seen that the *p*-values of the nozzle diameter, printing speed, extrusion pressure, and nozzle diameter × printing speed are all less than 0.05, indicating that these factors have a significant impact on filament width and height. Conversely, the *p*-value of the nondimensional nozzle height is greater than 0.05, indicating an absence of significant influence. This may be attributed to the small difference between the selected levels in the experiment. Within this narrow range, changing the parameters exerts minimal effects on the line width and height. Except for the single-factor and 2-way interaction effects listed in the figure, the impact of other 2-way interaction effects and multi-factor interactions is comparatively modest and not reflected in the figure.

**Figure 4:**
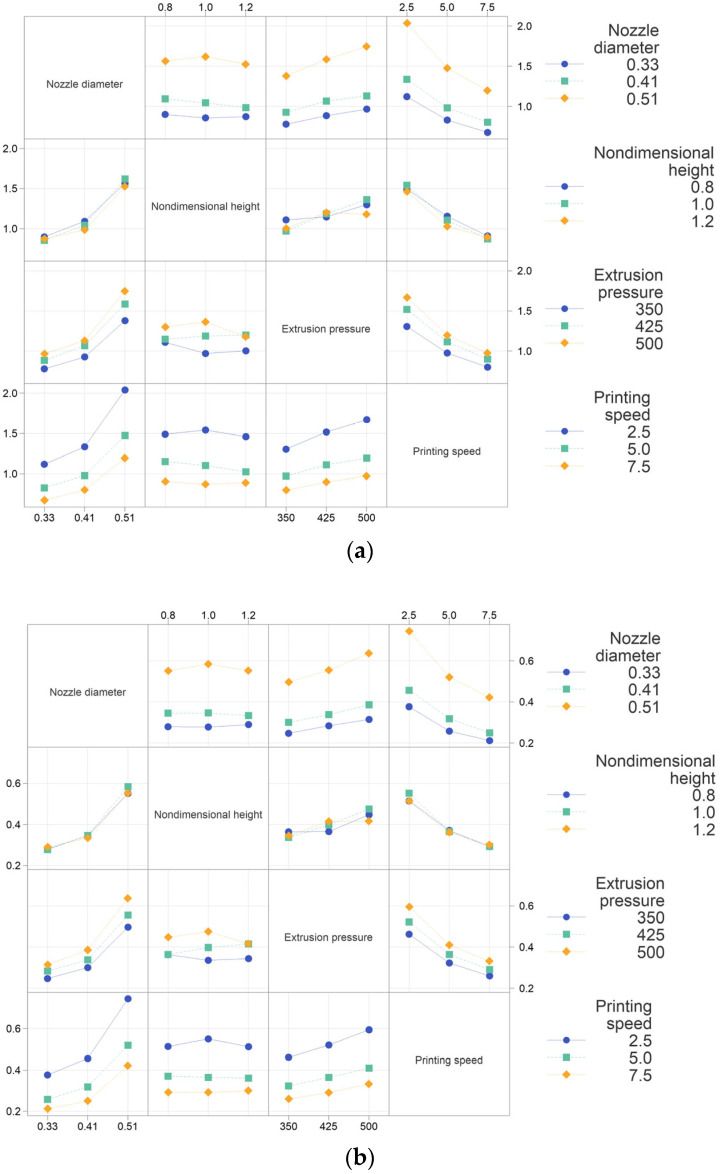
(**a**) Interaction plots of the filament width and (**b**) interaction plots of the filament height.

Figure 4a,b shows the factor interaction plot of filament width and height. There is a significant interaction between the nondimensional nozzle height and extrusion pressure; for changes in extrusion pressure, different nondimensional nozzle heights exhibit different trends. Specifically, when the nondimensional nozzle height is set at 0.8, an increase in extrusion pressure from 350 kPa to 425 kPa yields little rise in the filament width and height, which is different from the change at a nondimensional nozzle height of 1.0. This disparity may arise from the accumulation of printed materials, leading to the embedding of the nozzle tip at the lower nondimensional nozzle height of 0.8. Analogously, when the nondimensional nozzle height is 1.0 and extrusion pressure is elevated from 425 kPa to 500 kPa, an analogous circumstance occurs. That is to say, the difference in the nondimensional nozzle height and extrusion pressure can lead to different printing states. When selecting printing parameters, especially when the pressure is high and the printing speed is low, it is necessary to pay attention to the accumulation of materials on the substrate. If the nondimensional nozzle height is low, the nozzle may exert a squeezing effect on the material, resulting in differences in the forming performance compared to when there is no squeezing. Therefore, it should be avoided as much as possible or studied separately from the non-squeezing situation.

### 3.2. Regression Analysis

To specifically describe the relationship between the printing filament width/height and process parameters, we conducted a polynomial regression analysis. We utilize *A*, *B*, *C*, and *D* to represent nozzle diameter, nondimensional nozzle height, extrusion pressure, and printing speed, respectively, consistent with the abbreviations used in the ANOVA mentioned above. In the regression equation, four single factors *A*, *B*, *C*, and *D* were considered, along with their squared terms *A*^2^, *B*^2^, *C*^2^, and *D*^2^. Moreover, we integrate the double interaction terms *A* × *B*, *A* × *C*, and *A* × *D*, all of which exhibit a significant impact on the results. By regression fitting 70 sets of data, we obtained regression models for the line width and height, as shown in Equations (5) and (6):

*W* = 4.66 − 7.76 × *A* − 1.98 × *B* − 0.0084 × *C* − 0.1302 × *D* + 16.79 × *A*^2^ + 1.20 × *B*^2^ + 0.000011 × *C*^2^ + 0.02172 × *D*^2^ − 1.24 × *A* × *B* + 0.00384 × *A* × *C* − 0.473 × *A* × *D* (5)

*H* = 1.413 − 3.43 × *A* − 0.009 × *B* − 0.00307 × *C* − 0.0485 × *D* + 6.21 × *A*^2^ + 0.089 × *B*^2^ + 0.000003 × *C*^2^ + 0.00787 × *D*^2^ − 0.370 × *A* × *B* + 0.002481 × *A* × *C* − 0.1696 × *A* × *D* (6)

where *W* and *H* refer to the filament width and height.

**Figure 5:**
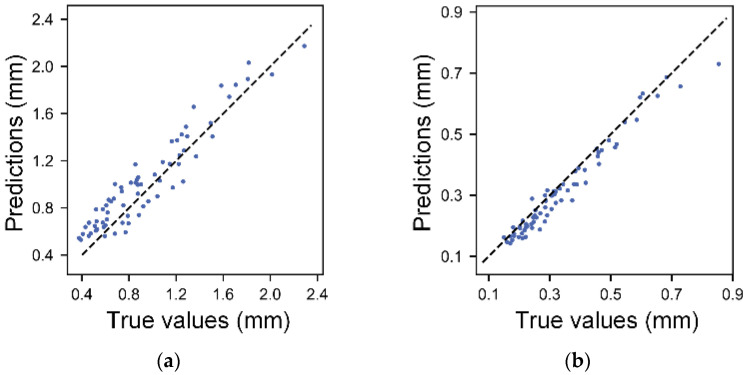
Scatter plots of the predicted values calculated by regression equation versus the experimental values: (**a**) filament width and (**b**) filament height.

The coefficient of determination *R*^2^ for the filament width and height are 0.8996 and 0.9641. Figure 5 illustrates the relationship between the predicted values of the regression equation and the true values. The data points that closely align with the dashed line indicate a strong agreement between the predicted and actual values. Obviously, the prediction accuracy for the filament height is notably higher, which aligns cohesively with the coefficient of determination outcomes. These polynomial regression models offer a more comprehensive perspective on the influence of the printing parameters over the filament width and height. This mathematical representation aids in achieving a deeper comprehension of this intricate relationship.

Furthermore, we conducted an analysis that considers the interaction between all two factors and establishes a polynomial regression equation containing complete quadratic terms. The coefficient of determination of the line width and height were 0.9044 and 0.9685, respectively. The results can be found in Equations (S1) and (S2) (Supplementary Materials). The fitting results containing the complete quadratic terms introduced three additional parameters, but the improvement in the regression coefficient was less than 0.5%. This further proves that the main effect factors we previously obtained from ANOVA dominate.

### 3.3. Machine Learning Result Analysis

Five machine learning models were built, and optimal hyperparameters were determined by 5-fold cross-validation. We used the average *RMSE* as the evaluation criterion. The selected hyperparameter values are as follows:

For the SVR model, the radial basis function (RBF) kernel was employed, with the penalty coefficient *C* 41.46, gamma 0.0127, and epsilon value 0.1487 for the line width prediction, and a penalty coefficient *C* 21,544, gamma 0.00038, and epsilon value 0.00464 for the line height prediction. For the BPNN model, the activation function used was the rectified linear unit (ReLU), with 50 and 25 hidden layers for the filament width prediction and a learning rate of 0.03; 80 and 40 hidden layers for the filament height model and a learning rate of 0.1. Both models were trained over 1000 iterations. For the DT model, the minimum number of samples required to split an internal node for line width prediction was set to seven, the minimum numbers per leaf for each leaf node was selected as three, and the hyperparameters for the line height prediction were set to five and two, respectively. In the RF model, the number of trees predicted by the line width, the maximum tree depth per tree, the minimum number of samples required to split an internal node, and the minimum numbers per leaf were set to 50, 5, 3, and 2, respectively. The four hyperparameters used for the line height prediction were 75, 24, 3, and 1, respectively. The sampling with the replacement method was then used. For the KNN model, the Manhattan distance was used, and inverse distance weight for each point was applied, which means the weight for each point is inversely proportional to their distance. The number of nearest neighbors used for the line width prediction was five, and the number of nearest neighbors used for the line height prediction was four.

After determining the hyperparameters, the machine learning models were trained, and both the training data and test data were predicted by the models. We calculated the *RMSE*, *MAE*, and *R*^2^, as shown in Table 4 and Table 5. For the prediction of filament width, the *R*^2^ of the five different algorithms on the test dataset ranged from 0.7734 to 0.9025, while the *R*^2^ of the filament height prediction ranged from 0.9205 to 0.9604. It is evident that the prediction accuracy of the filament height outperforms that of the filament width. We then made some comparisons to previously reported studies. Ali et al. [32] predicted the tensile strength and flexural strength of 3D-printed concrete materials by four machine learning methods, with *R*^2^ values ranging from 0.7253 to 0.8785. Wang et al. [30] predicted the drug loading efficiency of 3D-printed drugs in relation to material and process parameters, with *R*^2^ values ranging from 0.678 to 0.93. In contrast, the model established in this article has a similar performance for filament width prediction, while it performs more accurately with filament height prediction.

**Figure 6:**
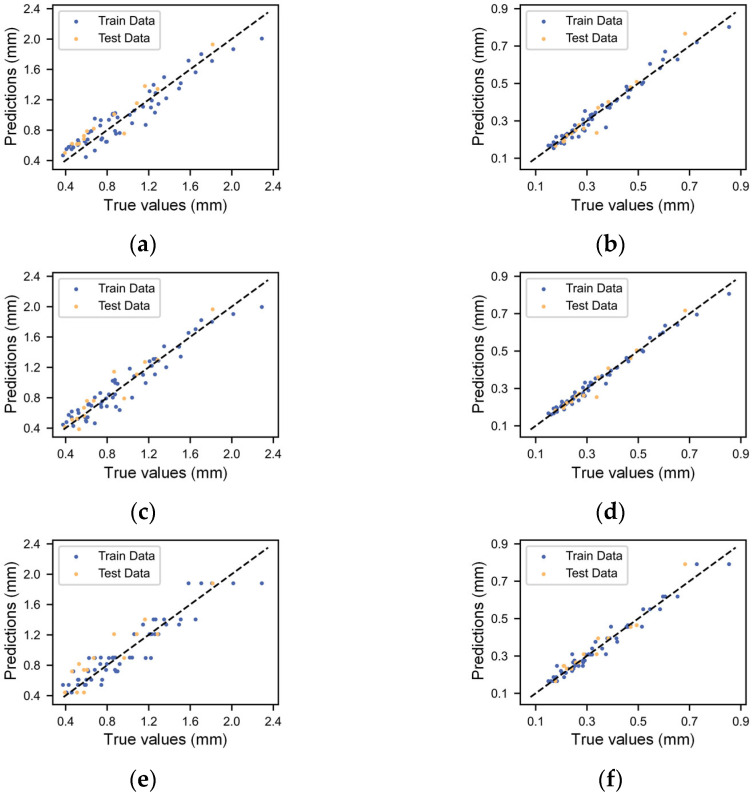

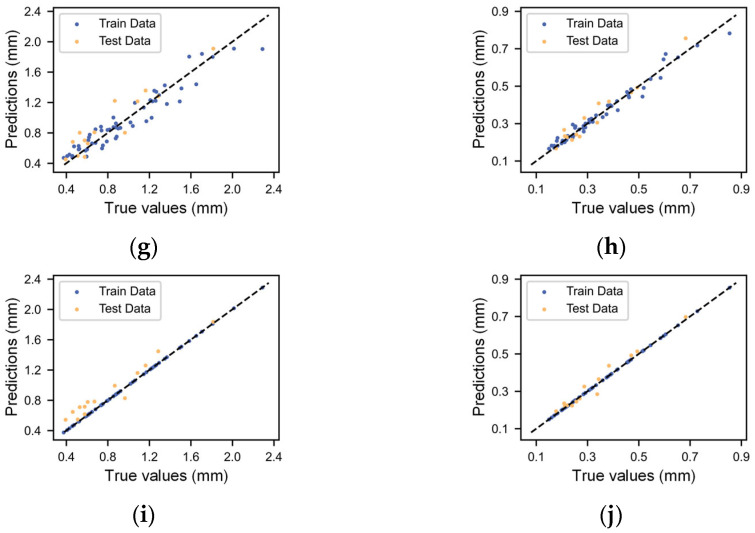
Scatter plots of the predicted values calculated by the different machine learning models versus the true values: (**a**) SVR width, (**b**) SVR height, (**c**) BP NN width, (**d**) BP NN height, (**e**) DT width, (**f**) DT height, (**g**) RF width, (**h**) RF height, (**i**) KNN width, and (**j**) KNN height.

Figure 6 shows the relationship between the predicted values and true values of the five machine learning methods. Combining the data in Table 4 and Table 5, it can be seen that the prediction performance of the BPNN and KNN methods is significantly higher than the other three methods. The neural network method excels at addressing the challenges posed by the non-smooth and non-linear features [46,52] and showed good prediction results in this study. The KNN method hinges on predicting the filament dimensions based on their proximity to processing parameters. Given that the variations in the printed line width and height tend to be relatively gradual within the ‘printability windows’, and our experiment maintains a fairly uniform distribution of process parameters, the KNN method yields commendable prediction results. The *R*^2^ of the training set is 1, which is mainly attributed to our model’s utilization of inverse distance weight weighting, that is, the weight of the nearest neighbor points is inversely proportional to their distance from the predicted data points [53]. In this case, the model overfits the training set because the closest sample to the training set data is itself, which has a large weight and presents a perfect prediction effect. The performance of the DT and RF models in predicting the results is not as good as the BPNN and KNN methods. The DT model employs a hierarchical structure and information entropy to create binary or multi-branched forks, resembling a tree-like structure [54]. The RF model is composed of DT as the basic unit and integrates a large number of DTs, obtaining results by averaging the predictions of each parallel tree through statistical analysis [55]. In this study, due to limited training data, the complexity and prediction accuracy of the DT or RF model were limited, especially in the filament width prediction, with an *R*^2^ of only around 0.8. In addition, the noticeable gap between the *R*^2^ of the two algorithms on the test set and the training set is significant, indicating the models’ weak generalization capabilities. Although the SVR model can perform well in solving nonlinear problems, its prediction results in this study are inferior to the prediction results of the BPNN and KNN methods.

### 3.4. Printing Process Parameter Analysis

**Figure 7:**
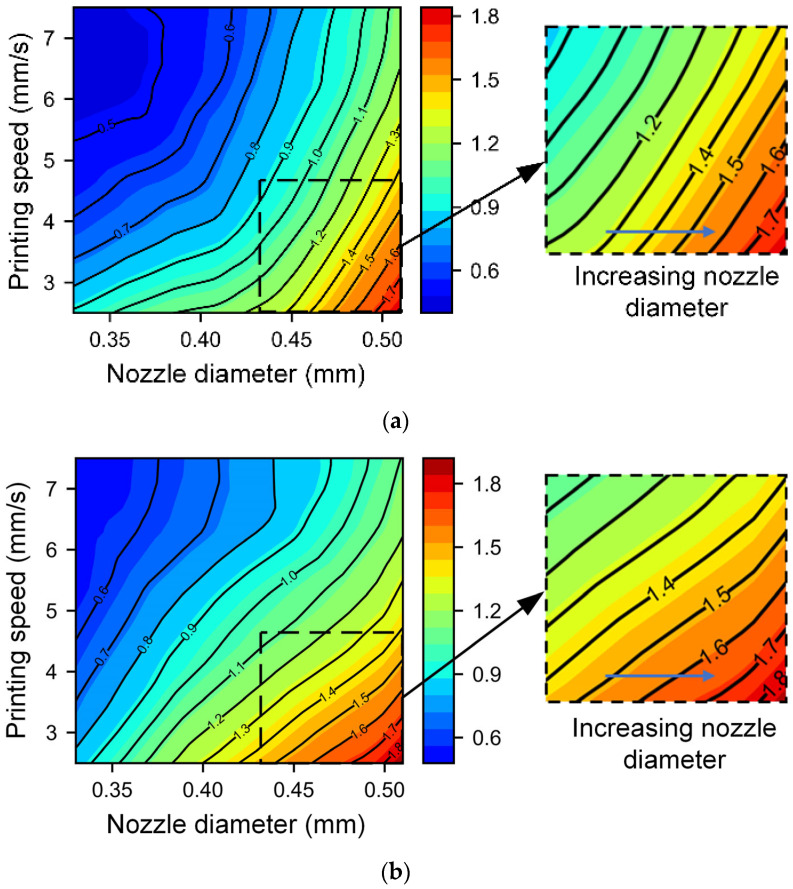
Contour map of the filament width with different nozzle diameters and printing speeds under different nondimensional nozzle heights: (**a**) nondimensional nozzle height of 0.85 and (**b**) nondimensional nozzle height of 1.15.

**Figure 8:**
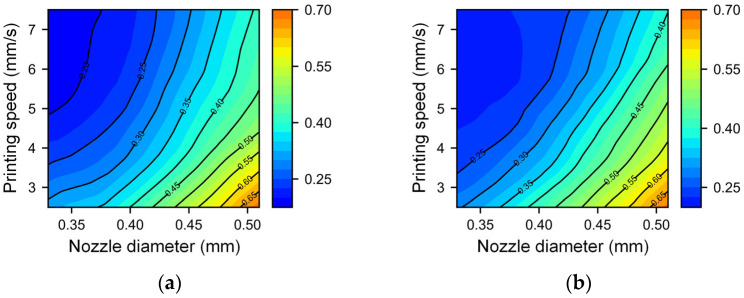
Contour map of the filament height with different nozzle diameters and printing speeds under different nondimensional nozzle heights: (**a**) nondimensional nozzle height of 0.85; (**b**) nondimensional nozzle height of 1.15.

The BPNN model performs the best of all the five models. Therefore, we utilize the BPNN model to further analyze the impact of the 3D printing process parameters. According to the DoE analysis results, the nozzle diameter and printing speed have the greatest impact on the filament width and height. Therefore, we choose these two process parameters as variables to draw contour maps for different combinations of other process parameters. Figure 7 and Figure 8 show the line width and height at different printing nozzle diameters (0.33 mm to 0.51 mm) and printing speeds (2.5 mm/s to 7.5 mm/s), with a fixed extrusion pressure of 420 kPa and nondimensional nozzle heights of 0.85 and 1.15. The trend of changes in the line width and height is similar under different printing heights. This consistency indicates that the effect of the nondimensional nozzle height on the line width and height is small, aligning with the DoE analysis results. When the nozzle diameter is small and speed is high (top left corner of each figure), the filament width and height are small and the contour lines are sparse, indicating that the size of the line structure is not very sensitive to changes in the nozzle diameter and printing speed. However, the filament width shows slightly different rules at low speeds and large nozzle diameters under different nondimensional nozzle heights. As shown in the local enlarged drawing of Figure 7a,b, for a low nozzle height, the filament width increases faster as the nozzle diameter increases (as shown by the blue arrow). This phenomenon could be attributed to the material experiencing squeezing effects at lower nozzle heights. A wider nozzle diameter corresponds to a larger squeezing area, resulting in a more significant increase in filament width. When the nozzle height is elevated, the squeezing effect decreases, and the influence of nozzle diameter is weakened. The nozzle height (or nondimensional nozzle height) is an important processing parameter in material extrusion. Han et al. [49] showed that different nondimensional nozzle heights can lead to various line states, including sawtooth, beeline, meandering, and discontinuous. Yuk and Zhao [50] conducted a more detailed study on various phenomena in the structure of printed lines through experiments. Jin et al. [29] similarly documented varied line structures at different nozzle heights. A smaller height might lead to uncontrollable width due to material over-deposition, while excessive height might cause delay time before material deposition, during which the gravity-induced dragging effect becomes significant and the material breaks into drops to form a broken filament, causing printing failure. They also found that in the optimal printing height range, the change in filament width is very small, which is consistent with the conclusion in this paper that the impact of the dimensionless nozzle height on filament width and height is small.

**Figure 9:**
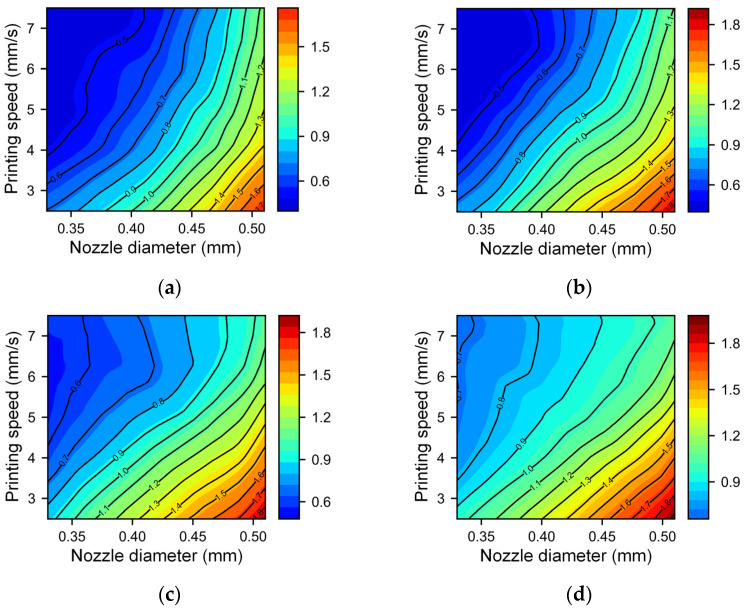
Contour map of the filament width with different nozzle diameters and printing speeds under different extrusion pressures: (**a**) 360 kPa, (**b**) 400 kPa, (**c**) 440 kPa, and (**d**) 480 kPa.

**Figure 10:**
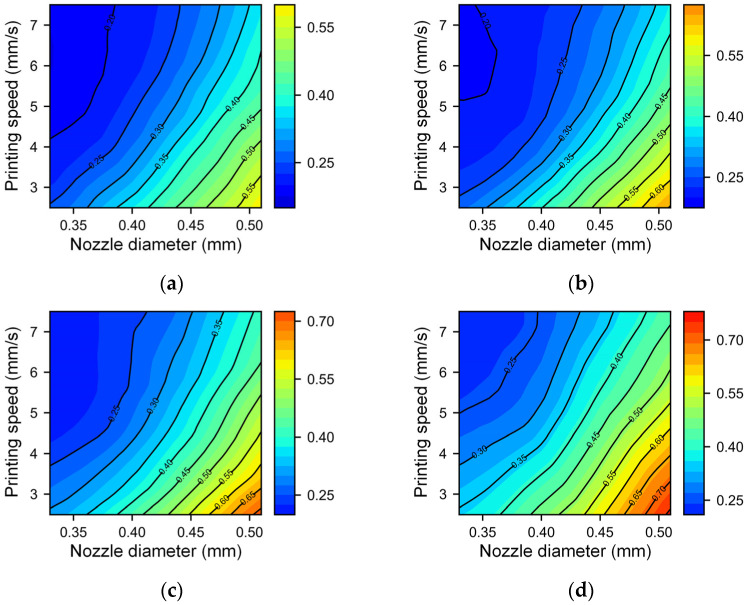
Contour map of the filament height with different nozzle diameters and printing speeds under different extrusion pressures: (**a**) 360 kPa, (**b**) 400 kPa, (**c**) 440 kPa, and (**d**) 480 kPa.

For the analysis of extrusion pressure, we systematically varied the extrusion pressures at 360 kPa, 400 kPa, 440 kPa, and 480 kPa. To reduce the potential impact of nozzle squeezing, we set the nondimensional nozzle height to 1.1, while the nozzle diameter and printing speed retained their prior ranges. Figure 9 and Figure 10 show the relationship between the filament width/height with different printing speeds and nozzle diameters under different pressures. It can be observed that as the extrusion pressure increases, the printing line width and height also increase. Regardless of the difference in extrusion pressure, the interplay between the nozzle diameter and printing speed remains remarkably consistent. In scenarios where the printing speed is high and the nozzle diameter is small (top left corner of each figure), the changes in the line width and height are relatively slow, but the shape of the contour line is intricate. This may be attributed to finer fibers being extruded which are disturbances from equipment vibrations, airflow, and other influences before deposition onto the substrate. Macroscopically, these manifest as a lack of regularity between the printing filament width and height and process parameters. The dimensions in this area are relatively small, which means that the printing resolution is high, so high-resolution printing requires strict control of disturbances. In the case of a low speed and large nozzle diameter (bottom right corner of each figure), the changes in the line width and height are more severe. As the printing speed decreases and the nozzle diameter increases, the contour lines gradually tend to be parallel, and the density gradually becomes consistent. In this area, the printed line width and height are relatively large, making it suitable for efficient printing.

**Figure 11:**
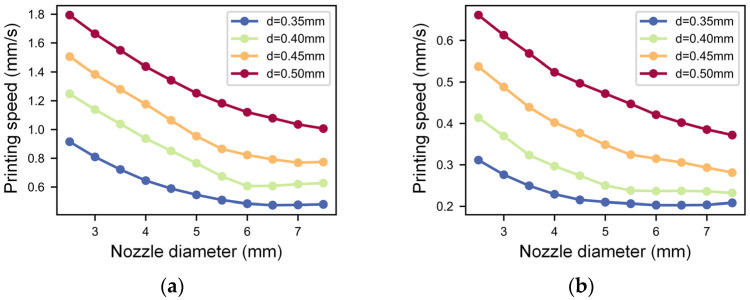
(**a**) Influence of extrusion pressure and nozzle diameter on the printed filament width and (**b**) influence of extrusion pressure and nozzle diameter on the printed filament height.

For the analysis of the nozzle diameter and printing speed, a nondimensional nozzle height of 1.1 and a moderate printing pressure of 420 kPa were selected. The trend of extrusion filament width and height with printing speed under different nozzle diameters is shown in Figure 11. The findings indicate that the line width and height decrease with increasing printing speed; the filament width and height are more sensitive to printing speed changes at low levels. While at high speeds, especially with small nozzle diameters, the line width and height trend changes are relatively stable, exhibiting minimal influence from heightened printing speeds. The line width and height increase with the increase in nozzle diameter, which is consistent with the results of the DoE analysis. Under identical printing speeds, uniform alterations in the nozzle diameter yield commensurate shifts in line width, revealing a muted interaction between the printing speed and nozzle diameter. The variation pattern of the line height is slightly different. Under constant printing speeds, when the nozzle diameter undergoes uniform variation, the augmentation in line height remains modest for small nozzle diameters. Conversely, a substantial increase in line height transpires with larger nozzle diameters. This means that the line height is more sensitive to changes in the nozzle diameter when the nozzle diameter is large. The filament width calculated by the BPNN model spans the range of 0.4747 mm to 1.7936 mm and the height ranges from 0.2026 mm to 0.6610 mm, when the nozzle diameter is between 0.35 mm–0.50 mm and the printing speed between 2.5 mm–7.5 mm/s, with a fixed printing pressure and nondimensional nozzle height. Compared with the filament width and height measured in our 70 experiments, these calculated ranges encapsulate 68.9% of the line width values and 65.1% of the line height values. Furthermore, the comparison with the printable range of other 2-way interaction parameter combinations is shown in Table S3. By comparison, it can be found that different parameter combinations of nozzle diameter and printing speed can print the largest range of line width and line height, which confirms the conclusion in the DoE analysis that printing speed and nozzle diameter play a dominant role in line width and line height.

## 4. Conclusions

This research investigated the relationship between the geometric dimensions of material extrusion 3D printing filaments and process parameters (nozzle diameter, nondimensional nozzle height, extrusion pressure, and printing speed). By employing a comprehensive approach encompassing DoE and ANOVA, the influence of various factors has been revealed. Machine learning models were established for predicting filament width and height, and the laws of various process parameters are analyzed based on the predicted results. The main conclusions of this research are summarized below:

(1) Based on the findings of the DoE and ANOVA results, the processing parameters affect the formation dimension in the order of nozzle diameter > printing speed > extrusion pressure > nondimensional nozzle height.
(2) Among the five machine learning prediction models trained, the BPNN and KNN methods have good performance, and the BPNN has the best coefficient of determination *R*^2^ for filament width and height prediction on the test set, being 0.9025 and 0.9604, respectively. All machine learning models have better filament height prediction results than width prediction. These models can be used for predicting printed structure dimensions, offering guidance for optimal parameter selection, informing the creation of 3D printing slices, and determining suitable layer sizes.
(3) Utilizing the BPNN model, the impact of printing process parameters on the line width and height was studied. The rules and reasons for the changes in filament width and height under different process parameter combinations were analyzed. The filament width and height are small and change slowly with a small nozzle diameter and high speed, making it suitable for high-resolution printing, while the filament width and height are large and change rapidly with a large nozzle diameter and low speed, making it suitable for high-efficiency printing. The interaction and coupling law between the nondimensional nozzle height and other factors are complex. Different nondimensional nozzle height settings may cause the extruded material to be stretched or squeezed. The material in the stretched state will lead to a thin filament, and the regularity of processing parameters on geometric size will not be strong. In addition, the nozzle diameter exhibits a significant impact on dimensions when the material is in a squeezed state. This study can be used to predict the size of printing filament structures, guide the selection of printing parameters, and determine the size of 3D printing layers.

## Figures and Tables

**Table 1:** List of the process parameters and respective sample numbers for the factorial design.

Number	Nozzle Diameter (mm)	Nondimensional Nozzle Height	Extrusion Pressure (kPa)	Printing Speed (mm/s)
1	0.33	0.8	350	5.0
2	0.33	0.8	425	2.5
3	0.33	0.8	500	7.5
4	0.33	1.0	350	2.5
5	0.33	1.0	425	7.5
6	0.33	1.0	500	5.0
7	0.33	1.2	350	7.5
8	0.33	1.2	425	5.0
9	0.33	1.2	500	2.5
10	0.41	0.8	350	7.5
11	0.41	0.8	425	5.0
12	0.41	0.8	500	2.5
13	0.41	1.0	350	5.0
14	0.41	1.0	425	2.5
15	0.41	1.0	500	7.5
16	0.41	1.2	350	2.5
17	0.41	1.2	425	7.5
18	0.41	1.2	500	5.0
19	0.51	0.8	350	2.5
20	0.51	0.8	425	7.5
21	0.51	0.8	500	5.0
22	0.51	1.0	350	7.5
23	0.51	1.0	425	5.0
24	0.51	1.0	500	2.5
25	0.51	1.2	350	5.0
26	0.51	1.2	425	2.5
27	0.51	1.2	500	7.5

**Table 2:** ANOVA results of filament width.

Factors	Degrees of Freedom	Sum of Squares	Mean Square	F-Value	*p*-Value
Nozzle diameter	2	2.36995	1.18497	529.79	<0.001
Nondimensional nozzle height	2	0.01681	0.00840	3.76	0.088
Extrusion pressure	2	0.29132	0.14566	65.12	<0.001
Printing speed	2	1.71309	0.85655	382.95	<0.001
Nozzle diameter × Nondimensional nozzle height	4	0.01710	0.00427	1.91	0.228
Nozzle diameter × Extrusion pressure	4	0.02985	0.00746	3.34	0.092
Nozzle diameter × Printing speed	4	0.13787	0.00224	15.41	0.003

**Table 3:** ANOVA results of filament height.

Factors	Degrees of Freedom	Sum of Squares	Mean Square	F-Value	*p*-Value
Nozzle diameter	2	0.392581	0.196291	569.85	<0.001
Nondimensional nozzle height	2	0.000719	0.000360	1.04	0.408
Extrusion pressure	2	0.042933	0.021466	62.32	<0.001
Printing speed	2	0.253553	0.126776	368.04	<0.001
Nozzle diameter × Nondimensional nozzle height	4	0.002018	0.000505	1.46	0.321
Nozzle diameter × Extrusion pressure	4	0.004460	0.001115	3.24	0.097
Nozzle diameter × Printing speed	4	0.021411	0.005353	15.54	0.003

**Table 4:** Statistical measures of different machine learning models for filament width prediction with the training and testing set.

ML Algorithms	Training Set			Testing Set		
	*RMSE*	*MAE*	*R* ^2^	*RMSE*	*MAE*	*R* ^2^
SVR	0.1381	0.1244	0.8969	0.1407	0.1329	0.8677
BP NN	0.1188	0.0955	0.9237	0.1209	0.0943	0.9025
DT	0.1483	0.1182	0.8812	0.1843	0.1597	0.7734
RF	0.1258	0.0999	0.9145	0.1671	0.1369	0.8139
KNN	0.0	0.0	1.0	0.1284	0.1168	0.8900

**Table 5:** Statistical measures of different machine learning models for filament height prediction with the training and testing set.

ML Algorithms	Training Set			Testing Set		
	*RMSE*	*MAE*	*R* ^2^	*RMSE*	*MAE*	*R* ^2^
SVR	0.0286	0.0203	0.9657	0.0382	0.0250	0.9205
BP NN	0.0186	0.0144	0.9855	0.0270	0.0171	0.9604
DT	0.0283	0.0210	0.9664	0.0377	0.0272	0.9227
RF	0.0250	0.0174	0.9739	0.0378	0.0306	0.9221
KNN	0.0	0.0	1.0	0.0270	0.224	0.9602

## Data Availability

The data presented in this study are available on request from the corresponding author.

## Supplementary Materials

The following supporting information can be downloaded at: https://www.mdpi.com/article/10.3390/mi14112091/s1, Table S1: List of the process parameters and respective sample number as the supplement of the DoE; Table S2: List of the average filament width and height for each 3D-printed sample; Table S3: The printed filament width and height range of different parameter combinations.

## References

[1] Cox J.R., Kipling I., Gibbons G.J. (2023). Ensuring supply chain integrity for material extrusion 3D printed polymer parts. Addit. Manuf..

[2] Hossain S.S., Jang S., Park S., Bae C.-J. (2023). Understanding ink design and printing dynamics of extrusion-based 3D printing: Defect-free dense piezoelectric ceramics. J. Manuf. Process..

[3] Koltsov S.I., Statsenko T.G., Morozova S.M. (2022). Modification of Commercial 3D Fused Deposition Modeling Printer for Extrusion Printing of Hydrogels. Polymers.

[4] Li W., Mille L.S., Robledo J.A., Uribe T., Huerta V., Zhang Y.S. (2020). Recent Advances in Formulating and Processing Biomaterial Inks for Vat Polymerization-Based 3D Printing. Adv. Healthc. Mater..

[5] Ng W.L., Xi H., Shkolnikov V., Goh G.L., Suntornnond R., Yeong W.Y. (2021). Controlling Droplet Impact Velocity and Droplet Volume: Key Factors to Achieving High Cell Viability in Sub-Nanoliter Droplet-based Bioprinting. Int. J. Bioprint..

[6] Ziaee M., Crane N.B. (2019). Binder jetting: A review of process, materials, and methods. Addit. Manuf..

[7] Shi X., Sun Y., Wang P., Ma Z., Liu H., Ning H. (2021). Compression properties and optimization design of SLM Ti6Al4V square pore tissue engineering scaffolds. Proc. Inst. Mech. Eng. Part H J. Eng. Med..

[8] Song B., Yu T., Jiang X., Xi W., Lin X., Ma Z., Wang Z. (2022). Development of the molten pool and solidification characterization in single bead multilayer direct energy deposition. Addit. Manuf..

[9] Qin J., Hu F., Liu Y., Witherell P., Wang C.C., Rosen D.W., Simpson T.W., Lu Y., Tang Q. (2022). Research and application of machine learning for additive manufacturing. Addit. Manuf..

[10] Luo Y., Sun W., Bao M., Zhu X., Ning C., Zhang W., Li Y., Zhang X. (2022). Process fundamentals and quality investigation in extrusion 3D printing of shear thinning materials: Extrusion process based on Nishihara model. Int. J. Adv. Manuf. Technol..

[11] Xu K., Li D., Shang E., Liu Y. (2022). A Heating-Assisted Direct Ink Writing Method for Preparation of PDMS Cellular Structure with High Manufacturing Fidelity. Polymers.

[12] Outrequin T.C.R., Gamonpilas C., Siriwatwechakul W., Sreearunothai P. (2023). Extrusion-based 3D printing of food biopolymers: A highlight on the important rheological parameters to reach printability. J. Food Eng..

[13] Zheng Q., Xie B., Xu Z., Wu H. (2023). A systematic printability study of direct ink writing towards high-resolution rapid manufacturing. Int. J. Extrem. Manuf..

[14] House A., Kuna A., Hastings D., Rodriguez N., Schoenitz M., Dreizin E.L., Guvendiren M. (2023). Effect of particle shape on rheology and printability of highly filled reactive inks for direct ink writing. Prog. Addit. Manuf..

[15] Wang J., Xu C., Yang S., Wang L., Xu M. (2023). Continuous and highly accurate multi-material extrusion-based bioprinting with optical coherence tomography imaging. Int. J. Bioprinting.

[16] Cheng L., Tang Q., Zhang Y., Cheng X., Miao A., Su J., Wu S., Niu F., Zhang L., Duan Y. (2023). Three-Dimensional Printed Multiresponsive Structures of Smart Hydrogel. 3D Print. Addit. Manuf..

[17] Schaffner M., Faber J.A., Pianegonda L., Rühs P.A., Coulter F., Studart A.R. (2018). 3D printing of robotic soft actuators with programmable bioinspired architectures. Nat. Commun..

[18] Zhang S., Xia Z., Liu Z., Wang Q., Yue Y., Huang J., Su B. (2023). Magnetic/conductive/elastic multi-material 3D-printed self-powered sensing gloves for underwater/smoke environmental Human-Computer Interaction. Chem. Eng. J..

[19] Wei F., Duan T., Yao L., Yang W. (2022). 3D printable and stretchable PVA-PAAm dual network hydrogel with conductivities for wearable sensors. J. Appl. Polym. Sci..

[20] Bie H., Chen H., Shan L., Tan C.Y., Al-Furjan M.S.H., Ramesh S., Gong Y., Liu Y.F., Zhou R.G., Yang W. (2023). 3D Printing and Performance Study of Porous Artificial Bone Based on HA-ZrO(2)-PVA Composites. Materials.

[21] Wang P., Sun Y., Li D., Ma Z., Zhang B., Diao L., Liu H. (2023). Extrusion-based 3D co-printing: Printing material design and novel workflow for fabricating patterned heterogeneous tissue structures. Mater. Des..

[22] Zhang J., Wu S., Wang Z., Chen Y., You H. (2023). Experimental Investigation of High-Viscosity Conductive Pastes and the Optimization of 3D Printing Parameters. Appl. Sci..

[23] Tian K., Bae J., Bakarich S.E., Yang C., Gately R.D., Spinks G.M., in het Panhuis M., Suo Z., Vlassak J.J. (2017). 3D Printing of Transparent and Conductive Heterogeneous Hydrogel–Elastomer Systems. Adv. Mater..

[24] Sevcik M.J., Bjerke G., Wilson F., Kline D.J., Morales R.C., Fletcher H.E., Guan K., Grapes M.D., Seetharaman S., Sullivan K.T. (2023). Extrusion parameter control optimization for DIW 3D printing using image analysis techniques. Prog. Addit. Manuf..

[25] Nijdam J.J., Agarwal D., Schon B.S. (2022). An experimental assessment of filament-extrusion models used in slicer software for 3D food-printing applications. J. Food Eng..

[26] Li M., Yu P., Guo Z., Liu Y., Zhao J. (2023). High-resolution and programmable line-morphologies of material-extrusion 3D printed self-leveling inks. Addit. Manuf..

[27] Udofia E.N., Zhou W. (2019). A Guiding Framework for Microextrusion Additive Manufacturing. J. Manuf. Sci. Eng..

[28] Seo H., Iwai H., Kishimoto M., Ding C., Saito M., Yoshida H. (2020). Microextrusion printing for increasing electrode–electrolyte interface in anode-supported solid oxide fuel cells. J. Power Sources.

[29] Jin Y., Zhao D., Huang Y. (2018). Study of extrudability and standoff distance effect during nanoclay-enabled direct printing. Bio-Design Manuf..

[30] Wang J., Heshmati Aghda N., Jiang J., Mridula Habib A., Ouyang D., Maniruzzaman M. (2022). 3D bioprinted microparticles: Optimizing loading efficiency using advanced DoE technique and machine learning modeling. Int. J. Pharm..

[31] Caputo M., Rashwan O., Waryoba D., McDade K. (2022). Surface texture and thermo-mechanical properties of material extruded and ironed polylactic acid. Addit. Manuf..

[32] Ali A., Riaz R.D., Malik U.J., Abbas S.B., Usman M., Shah M.U., Kim I.H., Hanif A., Faizan M. (2023). Machine Learning-Based Predictive Model for Tensile and Flexural Strength of 3D-Printed Concrete. Materials.

[33] Xue T., Wallin T.J., Menguc Y., Adriaenssens S., Chiaramonte M. (2020). Machine learning generative models for automatic design of multi-material 3D printed composite solids. Extreme Mech. Lett..

[34] Singh J., Singh J. (2021). A survey on machine learning-based malware detection in executable files. J. Syst. Archit..

[35] Kourou K., Exarchos T.P., Exarchos K.P., Karamouzis M.V., Fotiadis D.I. (2014). Machine learning applications in cancer prognosis and prediction. Comput. Struct. Biotechnol. J..

[36] Dabbagh S.R., Ozcan O., Tasoglu S. (2022). Machine learning-enabled optimization of extrusion-based 3D printing. Methods.

[37] Goh G.D., Sing S.L., Lim Y.F., Thong J.L.J., Peh Z.K., Mogali S.R., Yeong W.Y. (2021). Machine learning for 3D printed multi-materials tissue-mimicking anatomical models. Mater. Des..

[38] Sun X., Yue L., Yu L., Shao H., Peng X., Zhou K., Demoly F., Zhao R., Qi H.J. (2021). Machine Learning-Evolutionary Algorithm Enabled Design for 4D-Printed Active Composite Structures. Adv. Funct. Mater..

[39] Hamel C.M., Roach D.J., Long K.N., Demoly F., Dunn M.L., Qi H.J. (2019). Machine-learning based design of active composite structures for 4D printing. Smart Mater. Struct..

[40] McGregor D.J., Bimrose M.V., Shao C., Tawfick S., King W.P. (2022). Using machine learning to predict dimensions and qualify diverse part designs across multiple additive machines and materials. Addit. Manuf..

[41] Chen R., Bratten A., Rittenhouse J., Huang T., Jia W., Leu M.C., Wen H. (2022). Additive manufacturing of complexly shaped SiC with high density via extrusion-based technique—Effects of slurry thixotropic behavior and 3D printing parameters. Ceram. Int..

[42] Ma Y., Schutyser M.A., Boom R.M., Zhang L. (2021). Predicting the extrudability of complex food materials during 3D printing based on image analysis and gray-box data-driven modelling. Innov. Food Sci. Emerg. Technol..

[43] Jian G., Jiao Y., Meng Q., Shao H., Wang F., Wei Z. (2020). 3D BaTiO3 Flower Based Polymer Composites Exhibiting Excellent Piezoelectric Energy Harvesting Properties. Adv. Mater. Interfaces.

[44] Cai C., Chen T., Chen X., Zhang Y., Gong X., Wu C., Hu T. (2021). Enhanced Electromechanical Properties of Three-Phased Polydimethylsiloxane Nanocomposites via Surface Encapsulation of Barium Titanate and Multiwalled Carbon Nanotube with Polydopamine. Macromol. Mater. Eng..

[45] Zhu P., Yang W., Wang R., Gao S., Li B., Li Q. (2017). Direct Writing of Flexible Barium Titanate/Polydimethylsiloxane 3D Photonic Crystals with Mechanically Tunable Terahertz Properties. Adv. Opt. Mater..

[46] Suo S., Yang Y., Wang Z., Rao W.-F. (2023). The property palette: A rapid printing of performance-tunable blended polymers guided by artificial neural network. Appl. Mater. Today.

[47] Chicco D., Warrens M.J., Jurman G. (2021). The coefficient of determination R-squared is more informative than SMAPE, MAE, MAPE, MSE and RMSE in regression analysis evaluation. PeerJ Comput. Sci..

[48] Tang S., Yang L., Li G., Liu X., Fan Z. (2019). 3D printing of highly-loaded slurries via layered extrusion forming: Parameters optimization and control. Addit. Manuf..

[49] Han W., Gao W., Wang X. (2022). Implementation of printability for magneto-active soft materials based on programmed 3D printing technique. Smart Mater. Struct..

[50] Yuk H., Zhao X. (2018). A New 3D Printing Strategy by Harnessing Deformation, Instability, and Fracture of Viscoelastic Inks. Adv. Mater..

[51] Serdeczny M.P., Comminal R., Pedersen D.B., Spangenberg J. (2018). Experimental validation of a numerical model for the strand shape in material extrusion additive manufacturing. Addit. Manuf..

[52] Roach D.J., Rohskopf A., Hamel C.M., Reinholtz W.D., Bernstein R., Qi H.J., Cook A.W. (2021). Utilizing computer vision and artificial intelligence algorithms to predict and design the mechanical compression response of direct ink write 3D printed foam replacement structures. Addit. Manuf..

[53] ZGeler Z., Kurbalija V., Radovanović M., Ivanović M. (2015). Comparison of different weighting schemes for the kNN classifier on time-series data. Knowl. Inf. Syst..

[54] Hasan M., Islam M.M., Zarif M.I.I., Hashem M.M.A. (2019). Attack and anomaly detection in IoT sensors in IoT sites using machine learning approaches. Internet Things.

[55] Mehraein M., Mohanavelu A., Naganna S.R., Kulls C., Kisi O. (2022). Monthly Streamflow Prediction by Metaheuristic Regression Approaches Considering Satellite Precipitation Data. Water.

