# Supplementary material for: Material Extrusion Filament Width and Height Prediction via Design of Experiment and Machine Learning
Source: Micromachines (Basel). 2023 Nov 12;14(11):2091. doi: 10.3390/mi14112091 (PMC10673448; doi:10.3390/mi14112091)
Supplement: Supplementary file 1 [file micromachines-14-02091-s001.zip › micromachines-2682361-supplementary.pdf]

Supplementary Material

Material Extrusion Filament Width and Height Prediction via Design of Experiment and Machine Learning

Xiaoquan Shi [1], Yazhou Sun [1,*], Haiying Tian [1], Puthanveettil Madathil Abhilash [2], Xichun Luo [2] and Haitao Liu [1,*]

[1] Department of Mechanical Engineering and Automation, Harbin Institute of Technology, Harbin 150001, China; xiaoquanhit@163.com (X.S.); thy0567@163.com (H.T.)

[2] Centre for Precision Manufacturing, Department of Design, Manufacturing & Engineering Management, University of Strathclyde, Glasgow G1 1XJ, UK; abhilash.p-m@strath.ac.uk (P.M.A.); xichun.luo@strath.ac.uk (X.L.)

* Correspondence: sunyzh@hit.edu.cn (Y.S.); hthit@hit.edu.cn (H.L.)

Table S1. List of the process parameters and respective sample numbers as the supplement of the DoE.

Number	Nozzle diameter (mm)	Nondimensional nozzle height	Extrusion pressure (kPa)	Printing speed (mm/s)
1	0.33	0.8	350	5.0
2	0.33	0.8	425	2.5
3	0.33	0.8	500	7.5
4	0.33	1.0	350	2.5
5	0.33	1.0	425	7.5
6	0.33	1.0	500	5.0
7	0.33	1.2	350	7.5
8	0.33	1.2	425	5.0
9	0.33	1.2	500	2.5
10	0.41	0.8	350	7.5
11	0.41	0.8	425	5.0
12	0.41	0.8	500	2.5
13	0.41	1.0	350	5.0
14	0.41	1.0	425	2.5
15	0.41	1.0	500	7.5
16	0.41	1.2	350	2.5
17	0.41	1.2	425	7.5
18	0.41	1.2	500	5.0
19	0.51	0.8	350	2.5
20	0.51	0.8	425	7.5
21	0.51	0.8	500	5.0
22	0.51	1.0	350	7.5
23	0.51	1.0	425	5.0
24	0.51	1.0	500	2.5
25	0.51	1.2	350	5.0
26	0.51	1.2	425	2.5
27	0.51	1.2	500	7.5
28	0.51	1.2	350	7.5
29	0.41	1.0	425	7.5
30	0.33	1.1	350	2.5
31	0.51	1.2	500	5.0
32	0.33	0.8	390	6.3
33	0.51	1.0	500	6.3
34	0.41	1.0	350	7.5
35	0.41	0.8	390	3.8
36	0.41	1.1	390	6.3
37	0.33	1.0	425	5.0
38	0.41	1.1	390	7.5
39	0.33	0.8	460	6.3
40	0.51	1.2	460	2.5
41	0.33	1.0	460	2.5
42	0.41	1.0	350	6.3
43	0.41	0.8	460	7.5
44	0.33	0.9	460	6.3
45	0.41	1.2	390	7.5
46	0.51	1.1	350	7.5
47	0.41	0.9	460	5.0

48	0.33	0.8	390	7.5
49	0.33	1.0	390	2.5
50	0.51	0.9	425	7.5
51	0.41	1.0	460	7.5
52	0.41	0.8	425	3.8
53	0.33	1.2	350	6.3
54	0.51	1.1	390	2.5
55	0.33	1.1	350	3.8
56	0.41	0.9	350	5.0
57	0.41	1.2	350	7.5
58	0.33	1.1	390	6.3
59	0.51	0.9	460	6.3
60	0.33	0.8	500	2.5
61	0.41	1.1	425	3.8
62	0.33	0.9	460	2.5
63	0.33	0.8	500	3.8
64	0.33	1.1	390	5.0
65	0.51	0.9	390	5.0
66	0.51	0.9	350	2.5
67	0.41	1.1	460	6.3
68	0.51	0.8	390	5.0
69	0.41	0.9	425	3.8
70	0.33	0.9	500	7.5

Table S2. List of the average filament width and height for each 3D-printed sample.

Sample number	Average filament width (mm)	Average filament height (mm)	Sample number	Average filament width (mm)	Average filament height (mm)
1	0.7707	0.2213	36	0.5764	0.2396
2	1.1711	0.3738	37	0.5283	0.2089
3	0.7516	0.2422	38	0.5092	0.2211
4	0.9639	0.3377	39	0.5246	0.1984
5	0.6808	0.2108	40	1.8159	0.6829
6	0.9204	0.2840	41	0.6810	0.3152
7	0.5945	0.1830	42	0.5844	0.2024
8	0.7960	0.2677	43	0.6472	0.2458
9	1.2216	0.4180	44	0.5996	0.1699
10	0.7436	0.2168	45	0.4592	0.2259
11	1.0404	0.3058	46	1.2597	0.3886
12	1.4925	0.5144	47	0.7382	0.3203
13	0.8851	0.2915	48	0.4076	0.1601
14	1.3689	0.4618	49	0.6705	0.2871
15	0.8757	0.2853	50	1.0856	0.3833
16	1.1449	0.3937	51	0.5768	0.2563
17	0.7895	0.2470	52	0.8777	0.3138
18	1.0179	0.3590	53	0.3749	0.1496
19	1.8093	0.6532	54	1.7067	0.6046
20	1.2293	0.4150	55	0.4616	0.2113
21	1.6512	0.5850	56	0.6176	0.2513
22	1.0604	0.3794	57	0.4296	0.2026
23	1.5081	0.5194	58	0.3924	0.1770
24	2.2892	0.8541	59	1.2078	0.4594

25	1.2661	0.4575	60	0.8531	0.3331
26	2.0145	0.7283	61	0.8744	0.2922
27	1.2939	0.4697	62	0.8166	0.3065
28	0.8661	0.3436	63	0.7337	0.2526
29	0.6116	0.2227	64	0.4737	0.1830
30	0.6249	0.2424	65	1.1634	0.4704
31	1.3484	0.5461	66	1.5834	0.5964
32	0.4580	0.1703	67	0.6062	0.2691
33	1.2851	0.4937	68	1.2472	0.4542
34	0.5186	0.2178	69	0.8541	0.3195
35	0.9002	0.2845	70	0.5190	0.1801

Table S3. The printed filament width and height range of different parameter combinations.

Nozzle diameter (mm)	Nondimensional nozzle height	Extrusion pressure (kPa)	Printing speed (mm/s)	Filament width (mm)	Filament height (mm)
0.35 - 0.50	1.1	420	2.5 - 7.5	0.4747 - 1.7936	0.2026 - 0.6610
0.35 - 0.50	1.1	350 - 450	5	0.4798 - 1.3575	0.1901 - 0.5660
0.35 - 0.50	0.8 - 1.2	420	5	0.4051 - 1.3732	0.2048 - 0.4995
0.42	0.8 - 1.2	420	2.5 - 7.5	0.5311 - 1.4478	0.2382 - 0.4675
0.42	0.8 - 1.2	350 - 450	5	0.6633 - 1.2162	0.2605 - 0.3948
0.42	1.1	350 - 450	2.5 - 7.5	0.5368 - 1.4272	0.2217 - 0.5371

Equations (S1) and (S2):

$$W=5.18-7.69\times A-2.63\times B-0.01117\times C-0.014\times D+17.01\times A^2+1.08\times B^2+0.000012\times C^2+0.02207\times D^2-1.37\times A\times B+0.00352\times A\times C-0.481\times A\times D+0.00259\times B\times C-0.0288\times B\times D-0.000207\times C\times D \tag{S1}$$

$$H=1.460-3.554\times A-0.108\times B-0.00339\times C-0.0089\times D+6.407\times A^2+0.011\times B^2+0.000003\times C^2+0.00795\times D^2-0.408\times A\times B+0.00256\times A\times C-0.1788\times A\times D+0.000621\times B\times C-0.0006\times B\times D-0.000088\times C\times D \tag{S2}$$
